# A molecular precursor route to quaternary chalcogenide CFTS (Cu_2_FeSnS_4_) powders as potential solar absorber materials†

**DOI:** 10.1039/c9ra02926e

**Published:** 2019-08-05

**Authors:** Abdulaziz M. Alanazi, Firoz Alam, Abdelmajid Salhi, Mohamed Missous, Andrew G. Thomas, Paul O'Brien, David J. Lewis

**Affiliations:** School of Chemistry, University of Manchester Oxford Road Manchester M13 9PL UK; School of Materials, University of Manchester Oxford Road Manchester M13 9PL UK david.lewis-4@manchester.ac.uk; School of Electrical and Electronic Engineering, The University of Manchester Sackville Street Manchester M13 9PL UK; School of Chemistry, Islamic University Prince Naif Ibn Abdulaziz Rd Madinah 42351 Kingdom of Saudi Arabia

## Abstract

In the present work we report on the synthesis of tetragonal stannite Cu_2_FeSnS_4_ powders using a solvent free melt method using a mixture of Cu, Fe, and Sn(ii)/Sn(iv) *O*-ethylxanthates heated at different temperatures. The as-synthesized powders were characterized by powder X-ray diffraction (p-XRD), Raman spectroscopy, X-ray photoelectron spectroscopy (XPS), UV-Vis absorption spectroscopy, scanning electron microscopy (SEM) and energy dispersive X-ray (EDX) spectroscopy, which confirm the successful synthesis of stannite CFTS. Optical measurements show that Cu_2_FeSnS_4_ powders have visible light absorption onsets in the far red with direct band gap energies in the range 1.32–1.39 eV which are suitable for acting as efficient absorber layers in solar cells. Electronic characterisation of these materials deposited as thin films by spin coating show that they are p type semiconductors with respectable carrier mobilities of *ca.* 60 cm^2^ V^−1^ s^−1^ with carrier densities on the order of 10^14^ cm^−1^.

## Abstract 2

In the present work we report on the synthesis of a tetragonal phase of stannite Cu_2_FeSnS_4_ powder from Sn(ii) and Sn(iv) using a solvent free melt method using a mixture of Cu, Fe, Sn(ii)/Sn(iv) *O*-ethylxanthates.

## Graphical abstract

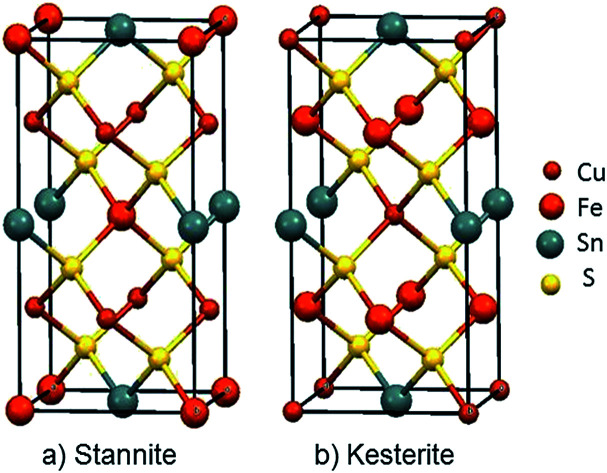

## Introduction

**Fig. 1:**
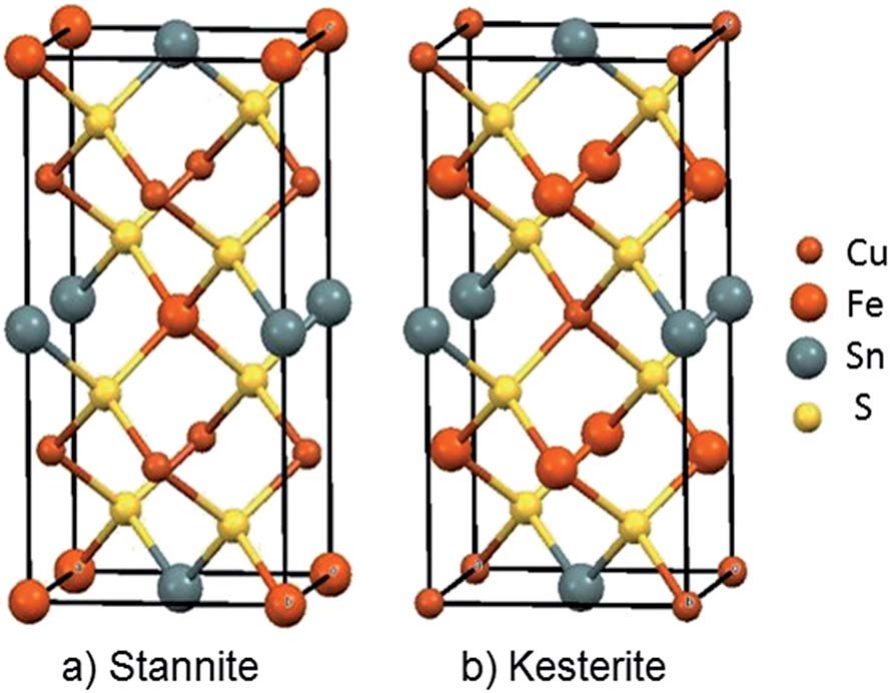
Unit cell representations of Cu_2_FeSnS_4_; (a) the Stannite type structure *a* = 5.449 Å; *c* = 10.726 Å, *α*, *β* and *γ* = 90°, ICDD: 0005838 (b) Kesterite type structure *a* = 5.434 Å; *c* = 10.856 Å, *α*, *β* and *γ* = 90° ICDD: 0005843.^23^

Among inorganic semiconductors, quaternary metal chalcogenides have attracted interest as light absorbers in photovoltaic applications.^1–8^ Copper iron tin sulphide (Cu_2_FeSnS_4_) has drawn considerable attention in photovoltaics because of its p-type conductivity, suitable band-gap 1.2–1.5 eV (Table 1) and high absorption coefficient (>10^4^ cm^−1^).^9–12^ The structure of Cu_2_FeSnS_4_ is similar to the zinc blende structure. Structures are adopted which depend on the configuration of the tetrahedral holes which are called stannite (CFTS) and kesterite (CZTS), respectively.^13^ Stannite is tetragonal with unit-cell parameters *a* = 5.449 Å, *c* = 10.726 Å with a space group *I*4̄2*m* as shown in Fig. 1(a), whilst kesterite is tetragonal with unit-cell parameters *a* = 5.434 Å, *c* = 10.856 Å (Fig. 1(b)) with a space group *I*4̄2*m*.^13^ Both phases consist of inexpensive, non-toxic and earth-abundant materials.

**Table 1:** Reported band gaps of CFTS nanomaterials prepared by different methods

Method	Band gap (eV)	Reference
Hot-injection	1.28	28
Solvothermal	1.33	9
Microwave irradiation	1.71	32
Electrospinning	1.24	36
Liquid reflux	1.32	37
Solution-based	1.46	38

Current commercialized thin film solar cells technologies such as CdTe and Cu_2_InGaS_4_ (CIGS) have commonly used elements which are expensive, rare and toxic such as In, Ga and Cd.^14^ Therefore, the development of low-cost, nontoxic and environmental friendly alternatives are needed to make high-efficiency solar cells. Copper based quaternary chalcogenides such as Cu_2_ZnSnS_4_ (CZTS) and Cu_2_ZnSnSe_4_ (CZTSe) have been used as solar absorber materials in thin film solar cells.^15,16^ One of the challenges in the synthesis of CZTSSe materials is to obtain pure and stoichiometric kesteritic materials as the optoelectronic properties are sensitive to in particular the Cu and Zn ratios.^10,17–22^ One of the alternatives to CZTS is to Cu_2_FeSnS_4_ (CFTS) which has been used as an Pt-free counter electrode in dye-sensitized solar cells (DSSCs) as well as an absorber material in thin film solar cells.^23^

CZTS and CFTS have suitable optical band gaps of around 1.4 eV and good absorption coefficients (typically *α* > 10^4^ cm^−1^) in the visible spectral range which is comparable to CIGS materials, making them favourable candidates for photovoltaic applications.^24,25^ Hence, CZTS and CFTS thin film solar cells have reached power conversion efficiencies of 12.6% and 8.03%, respectively, where these materials are used as part of the absorber layer.^26^ In addition CZTS and CFTS show p-type conductivities which can be useful for pairing to n-type materials in cell architectures.^27^

Up to now range of methods have been reported for the synthesis of CFTS materials of different shapes and sizes.^28–30^ Some reports focus on developing solution based processes as an alternate to vacuum deposition. This offers the advantage of high productivity and low processing temperatures.^31^ Other methods such as solvothermal processing,^29^ hot injection^28^ and microwave irradiation^32^ have been used for solution based synthesis of CFTS. However, the solvothermal and hot injection processes have restrictive conditions, often give low yields, use toxic chemicals (ethylenediamine and oleylamine) and require heat treatment for 18–24 h, centrifugation and vacuum drying.

Thus, it is necessary to design inexpensive approaches for synthesis of CFTS materials. In this paper, we produce CFTS from direct thermal decomposition of metal xanthate precursors. To the best of our knowledge, the synthesis of CFTS powders using solvent free thermolysis has not been reported so far. The method which we propose has been used to prepare CFTS powders in large quantities. The technique is straight forward, solvent free, inexpensive and single step utilizing single source precursors (SSPs).^33–35^ Here, we use metal xanthate precursors because their decomposition happens at a lower temperature and the by-products are gaseous.^34,35,39^

## Experimental

### Materials

Tin(ii) chloride (99.9%), tin(iv) chloride (98%), carbon disulphide (99.9%), iron(iii) chloride (97%), copper(ii) sulphate (98%), chloroform (99.8%), hexane (97%), toluene (99.7%) and ethanol (99.8%) were purchased from Sigma-Aldrich or Alfa Aesar and used as received.

A Phillips X-PERT PRO with Cu Kα incident beam (*λ* = 1.54059 Å) was used to record X-ray diffraction patterns. The samples were scanned in the 2*θ* range of 10° to 80° for a period of 1 h. Scanning electron microscopy (SEM) was carried out using a Philips XL 30FEG. The voltage used was 40 kV. Carbon coating was carried out using an Edwards E306A coating unit. EDX spectroscopy (Philips EDAX DX4 X-ray micro-analyser SEM) was used to determine elemental composition as well used for elemental mapping in order to know the spatial distribution of elements in the sample. The optical properties of the CFTS powders were characterized by UV-Vis-NIR absorption spectroscopy recorded on a Shimadzu UV-1800. Raman spectra were collected using a Renishaw 1000 Micro-Raman system equipped with a 50× objective and a 514 nm laser. X-ray photoelectron spectroscopy (XPS) measurements were performed using either a Kratos Axis Ultra or SPECS XPS instrument. Both facilities are equipped with monochromated Al Kα X-ray sources with a photon energy of 1486.6 eV. Emitted photoelectrons were collected using either a 165 mm hemispherical energy analyser (Kratos) or a 150 mm hemispherical energy analyser (Phoibos 150 SPECS), respectively. The peaks were calibrated through referencing C 1s to 284.8 eV. Infrared spectra were recorded on a Specac single reflectance ATR instrument (4000–400 cm^−1^, resolution 4 cm^−1^). Melting points were determined using a Barloworld SMP10 device and the elemental analyses of complexes were done using a Flash 2000 Thermo Scientific elemental analyser. Thermogravimetric analysis (TGA) was performed using a Mettler Toledo TGA/DSC 1 system under an atmosphere of dry nitrogen.

### Synthesis of metal xanthate complexes

#### Synthesis of potassium ethylxanthate

The synthesis of the potassium ethylxanthate was conducted in the following way. Potassium hydroxide (11.29 g, 0.2 mmol) was dissolved in ethanol (75 ml) and cooled in an ice bath. Carbon disulphide (15.32 g, 12.16 ml, 0.2 mmol) was added dropwise while stirring. The ethanol was evaporated at room temperature to obtain the product, 71.8% yield.

#### Synthesis of bis (*O*-ethylxanthato) copper(ii)

The synthesis of [Cu(S_2_COEt)_2_] was carried out according to the literature.^40^ Briefly, an aqueous solution of potassium ethylxanthate (1.596 g, 9.9 mmol) and CuSO_4_·5H_2_O (1.242 g, 4.9 mmol) mixed at room temperature while stirring and the stirring was continue for 60 minutes. Orange precipitate was obtained and washed with deionised water. The precipitate was filtered, and then product was finally dried in a vacuum oven overnight at room temperature. Yield: 87%. Melting point: 187 °C. Elemental analysis: calc. (%): C, 23.58; H, 3.30; S, 41.85; Cu, 20.80. Found (%): C, 23.67; H, 3.21; S, 41.39; Cu, 21.09. IR (*ν*_max_/cm^−1^): 2979.63–2932.92 (w), 1462.34–1367.20 (s), 1239.20 (s), 1119.11 (s), 846 (w).

#### Synthesis of (*O*-ethylxanthato) copper(i) triphenylphosphine

The synthesis of [(Ph_3_P)_2_CuS_2_COEt] was carried out by following the literature.^41^ A mixture of triphenylphosphine (2.09 g, 0.008 mol) and CuCl (0.40 g, 0.0040 mol) was dissolved in 40 ml of chloroform and later it was added to the potassium ethylxanthate (0.641 g, 0.0040 mol) that was dissolved in 40 ml of chloroform. After stirring for 1 h at room temperature a white precipitate was obtained. The precipitate was filtered to obtain a clear yellow solution. At −20 °C the yellow crystals of *O*-ethylxanthato copper(i) triphenylphosphine was obtained. Yield: 85%. Melting point: 185–191 °C. Elemental analysis: calc. (%): C, 66.1; H, 4.97; S, 9.02; P, 8.74; Cu, 8.96. Found (%): C, 65.7; H, 5.08; S, 8.77; P, 8.44; Cu, 8.74. IR (*ν*_max_/cm^−1^): 3048 (w), 2992 (w), 1478 (m) 1433 (m), 1290 (s), 1142 (m), 1041 (m), 1009 (s), 849.5 (s), 740.8 (m), 617.7 (s), 559.2 (s).

#### Synthesis of tris (*O*-ethylxanthato) iron(iii)

The synthesis of [Fe(S_2_COEt)_3_] was carried out by following the literature.^42^ Briefly, an aqueous solution of potassium ethylxanthate (1.596 g, 9.9 mmol) and an aqueous solution of FeCl_3_ (0.538 g, 3.3 mmol) mixed at room temperature while stirring and the stirring was continue for 60 minutes. The black precipitates was obtained and washed with deionised water. The precipitate was filtered using Whatman paper, and the product was finally dried in a vacuum oven overnight at room temperature. Yield: 85%. Melting point: 118 °C. Elemental analysis: calc. (%): C, 25.79; H, 3.61; S, 45.81; Fe, 13.34. Found (%): C, 25.59; H, 3.35; S, 45.36; Fe, 12.70. IR (*ν*_max_/cm^−1^): 2987.63–2979.92 (w), 1458.55–1425.36 (s), 1233.18 (s), 1059.25 (s), 856 (w).

#### Synthesis of bis (*O*-ethylxanthato) tin(ii)

The synthesis of [Sn(S_2_COEt)_2_] was carried out by following the literature.^43^ Briefly, tin(ii) ethylxanthate was produced by adding an aqueous solution of K(S_2_COEt) (5 g, 31.1 mmol) into an aqueous solution of tin(ii) chloride (2.95 g, 15.5 mmol) in 50 ml deionised water while stirring and the stirring was continue for 60 minutes that results in a black precipitates. The precipitate was filtered using Whatman paper, and the product was finally dried in a vacuum oven overnight at room temperature. Yield: 87.5%. Melting point: 47 °C. Elemental analysis: calc. (%): C, 19.98; H, 2.79; S, 35.45; Sn, 32.91. Found (%): C, 20.04; H, 2.72; S, 35.19; Sn, 32.87 IR (*ν*_max_/cm^−1^): 2977.24–2935.84 (w), 1462.82–1364.91 (s), 1272.76 (s), 1113.75 (s), 860 (w).

#### Synthesis of tetrakis (*O*-ethylxanthato) tin(iv)

The synthesis of [Sn(S_2_COEt)_4_] was carried out using a technique that was modified from literature.^44^ Briefly, SnCl_4_ (1 g, 3.8 mmol) was dissolved in 50 ml of toluene and added drop by drop to the potassium ethylxanthate (2.5 g, 15.3 mmol) in toluene at room temperature. The reaction mixture was stirred for 60 minutes then precursor solution was evaporated under reduced pressure and then oily residue was shaken by adding 50 ml of hexane. The yellow crystals of [Sn(S_2_COEt)_4_] were extracted from the solution. Yield: 91.3%. Melting point = 73 °C. Elemental analysis: calc. (%): C, 23.91; H, 3.34; S, 42.43; Sn, 19.69. Found (%): C, 23.98; H, 3.29; S, 42.01; Sn, 20.11. IR (*ν*_max_/cm^−1^): 2987.29 (w), 1462.48–1365.84 (s), 1247.83 (s), 1025.47 (s), 860 (w).

### Synthesis of CFTS powders

For the synthesis of CFTS powders, 2 mmol copper(ii) ethylxanthate, 1 mmol iron(iii) ethylxanthate and 1 mmol tin(ii) or tin(iv) ethylxanthate were mixed together. Then the mixture was heated in a furnace at either 250 °C, 350 °C and 450 °C for 1 h under a nitrogen atmosphere. The CFTS powders produced were allowed to cool-down to room temperature in the inert atmosphere. The CFTS powder synthesised using Sn(ii) and Sn(iv) are named as (1) and (2), respectively.

In addition to the synthesis of CFTS powders, we also deposited the CFTS thin films using spin coating technique from Sn(ii) and Sn(iv), which are named as (3) and (4), respectively. Full details on the synthesis and characterisation of these thin film samples can be found in the ESI.†

## Results and discussion

### Thermogravimetric analysis (TGA) of precursors

**Fig. 2:**
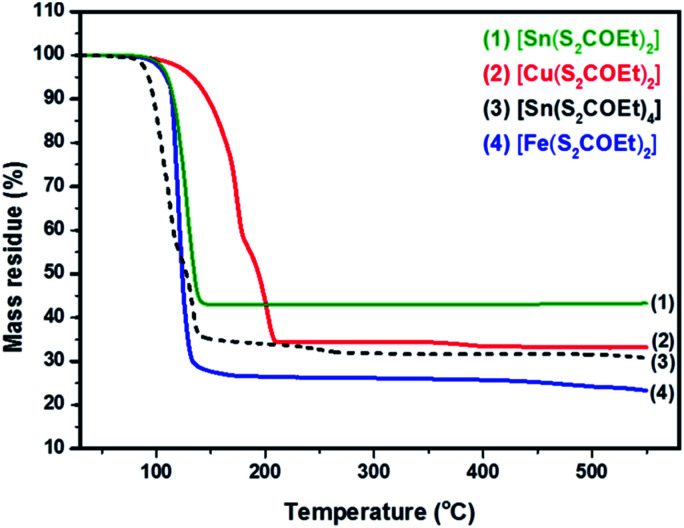
Thermogravimetric analysis of [Cu(S_2_COEt)_2_] (red colour), [Fe(S_2_COEt)_3_] (blue colour), [Sn(S_2_COEt)_2_] (green colour) and [Sn(S_2_COEt)_4_] (black colour) precursors.

The synthesis of [Cu(S_2_COEt)_2_], [Fe(S_2_COEt)_3_], [Sn(S_2_COEt)_2_] and [Sn(S_2_COEt)_4_] complexes were performed and their suitability for melt reactions was measured through thermal stability measurement in a nitrogen atmosphere. Fig. 2 shows the TGA profiles of [Sn(S_2_COEt)_2_], [Cu(S_2_COEt)_2_], [Sn(S_2_COEt)_4_] and [Fe(S_2_COEt)_3_], respectively. The [Cu(S_2_COEt)_2_] and [Sn(S_2_COEt)_4_] complexes display a two-step decomposition pattern. In the case of the [Cu(S_2_COEt)_2_] precursor, the mass residue obtained from the TGA profiles for the first decomposition stage (58%) agreed with the theoretical value calculated for the removal of one molecule of xanthate and half from another one (58%). While in the second step, there is a mass loss of 31% in the temperature range of 200 to 450 °C that agrees with theoretical value (31%) for production of CuS. In the case of [Sn(S_2_COEt)_4_] the first step involves a degradation of the mass loss 59% in the temperatures range of 45 to 120 °C obtained from the TGA profile, which corresponds and agreed with the theoretical value calculated for the removal of three molecules of xanthate (60%), and the final decomposition residue obtained after 150 °C was found to be SnS_2_ which is almost in conformity with the mass loss data obtained from the TGA profile (32%) and the theoretical value (33%). In contrast, the [Sn(S_2_COEt)_2_] and [Fe(S_2_COEt)_3_] precursor complexes have a single step decomposition. The single step decomposition of [Sn(S_2_COEt)_2_] occurred in the temperature range of 304–396 °C with a mass loss of 42% and for [Fe(S_2_COEt)_3_] is 27% in the temperature range of 73–400 °C which is in good agreement with the theoretical values of SnS (42%) and FeS_2_ (29%), respectively. A number of other authors have observed these typical weight loss steps with metal xanthates in their decomposition to corresponding metal sulfides.^35,39^ For instance, Almanqur *et al.*, have successfully synthesised a series of iron alkyl xanthate complexes to deposit iron sulphide thin films and nanostructures using the spin coating and the solvent free pyrolysis methods. The TGA profiles of these complexes showed approximately the same with a rapid residue loss within the temperature range of 120 to 300 °C, and final step occurred between 320 to 500 °C. All complexes showed the final solid residue amounts that matched with the calculated values for FeS_2_ or FeS.^34^

Al-Shakban *et al.*, have synthesised SnS thin films from diphenyltin bis(iso-butylxanthate) complexes using aerosol-assisted chemical vapor deposition (AACVD). The TGA profile of this complex showed two-step decomposition, the first step of which involves elimination of the alkyl groups, followed by carbonyl sulfide (SCO). Then, the final step may involve the loss of another carbonyl sulfide.^45^

### Bulk structural characterisation of CFTS powders

**Fig. 3:**
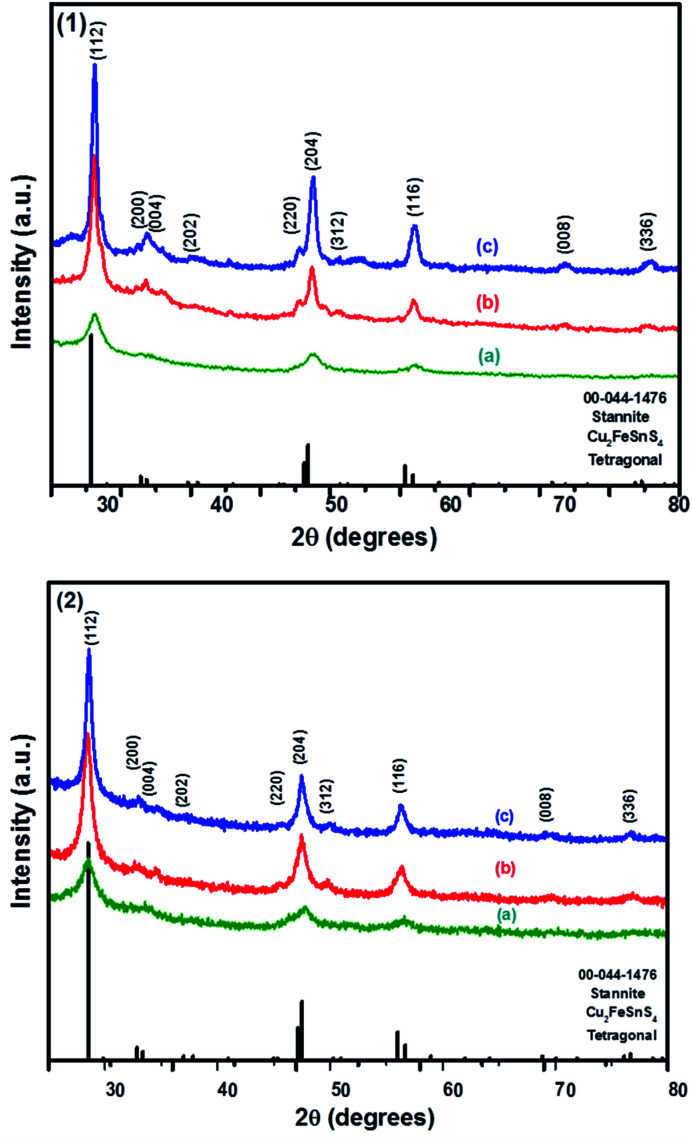
The p-XRD patterns of Cu_2_FeSnS_4_ powders (1) and (2) synthesised at (a) 250 °C; (b) 350 °C and (c) 450 °C for 1 h.

The powder XRD patterns of CFTS synthesized at different temperatures using Sn(ii) and Sn(iv) precursors are shown in Fig. 3. The diffraction peaks observed at 2*θ* values of 28.50°, 32.85°, 33.36°, 36.97°, 47.15°, 47.50°, 50.93°, 56.66°, 70.04° and 76.69° correspond to the (112), (200), (004), (202), (220), (204), (301), (116), (008) and (316) planes of tetragonal stannite, respectively.

The calculated lattice parameters for powder (1) and (2) are *a* = 5.4501 Å, *c* = 10.7468 and *a* = 5.4467 Å, *c* = 10.7510 Å, respectively which are in good agreement with the reported literature values for CFTS.^29^ The XRD peak intensities increased with increasing the temperature without affecting the phase of the powder. The average domain size of both powder (1) and (2) are approximately 13 nm calculated using Scherrer's equation. The comparison between kesterite and stannite with experimentally determined and calculated values is represented by the tetragonal distortion (deviation of the *c*/2*a* ratio from 1, where, *c* and *a* are the lattice parameters). The tetragonal distortion parameter is important for the resulting electronic structure of the material,^46^ for example, strong deviations away from the ideal structure caused by a changes in crystal field can lead to non-degenerate valence band maxima.^47–49^

In kesterite, the *c*/2*a* ratio has been reported to be greater than 1 in a neutron diffraction study done on powder samples.^46^ However, in stannite this ratio has been reported to be less than 1, as estimated using XRD studies. In our study, the ratio of stannite CFTS was determined to be 0.99, which is slightly less than 1 and thus this value is in good agreement with the values in the literature.^13,46^

**Fig. 4:**
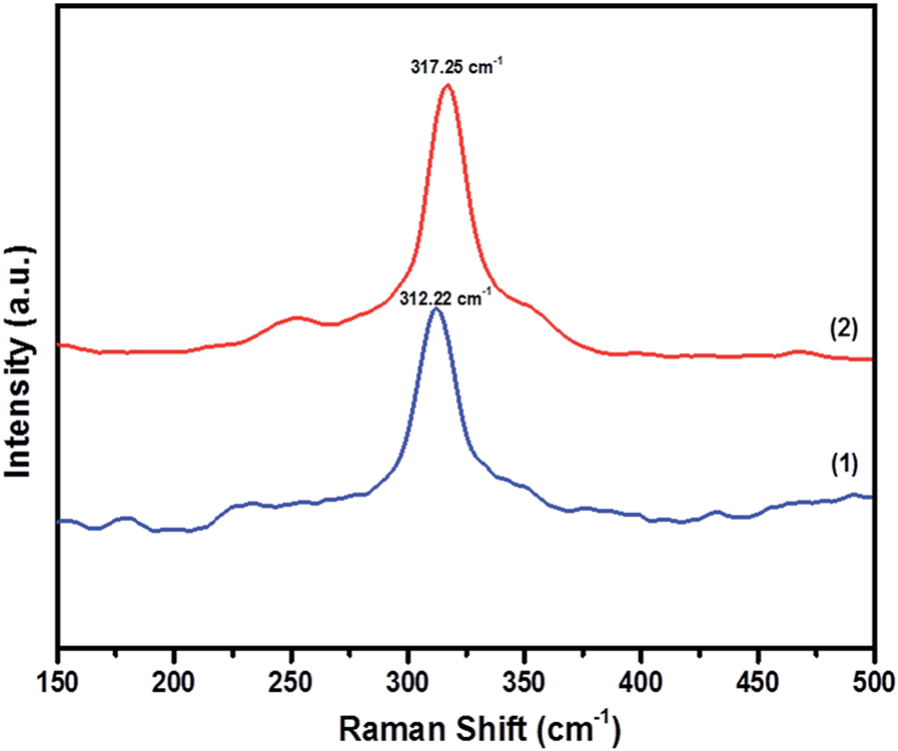
The Raman spectra of Cu_2_FeSnS_4_ powders (1) and (2) synthesized at a temperature of 450 °C for 1 h.

In order to prove the pristine nature of the synthesized powders and to rule out the existence of secondary phases that were not distinguished by the XRD, Raman spectroscopy was performed. Fig. 4 shows the Raman spectra of CFTS, which exhibits a large peak at 312.22 cm^−1^ and 317.25 cm^−1^ corresponding to tetragonal CFTS in both (1) and (2), respectively. It is reported in the literature that this is the A_1_ symmetric vibrational motion of sulphur atoms in CFTS.^50–52^

We also note the absence of Raman peaks corresponding to FeS (214 and 282 cm^−1^) and Cu_2_SnS_3_ (267, 303 and 356 cm^−1^) which are common contaminants of CFTS,^53^ and is consistent with the XRD patterns of the powders being a single crystalline phase (Fig. 3).

**Fig. 5:**
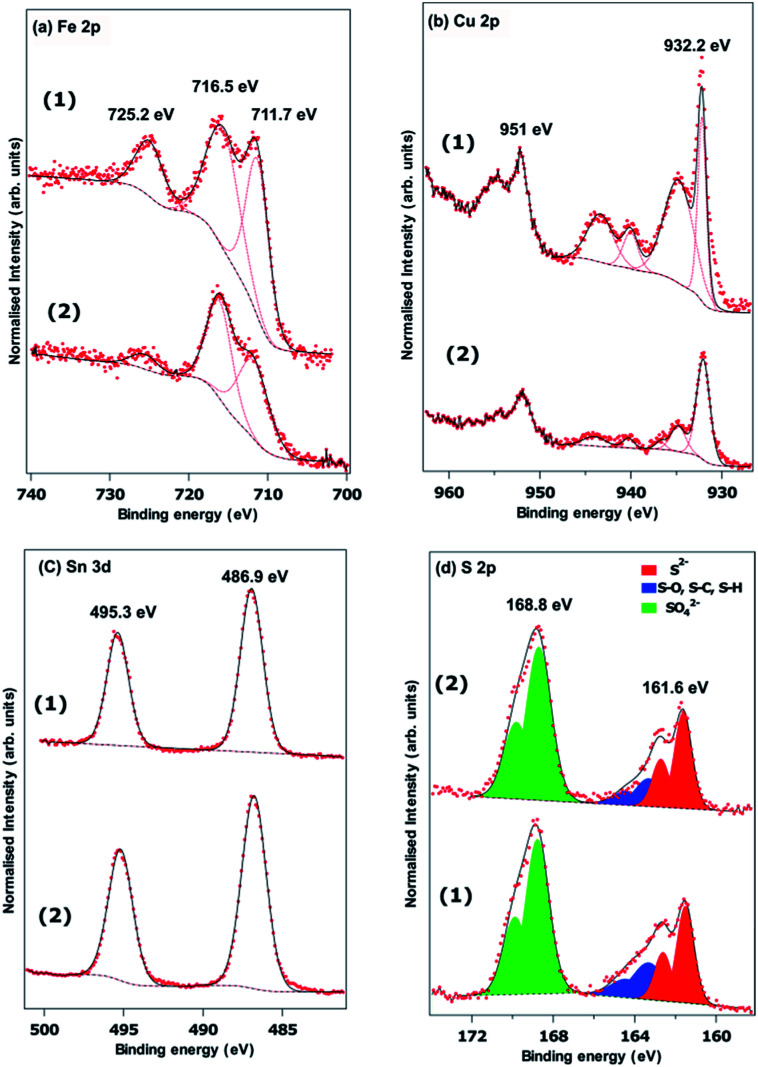
XPS spectra of Cu_2_FeSnS_4_ powders (1) and (2) synthesized at a temperature of 450 °C for 1 h: (a) Fe 2p, (b) Cu 2p, (c) Sn 3d and (d) S 2p.

**Fig. 6:**
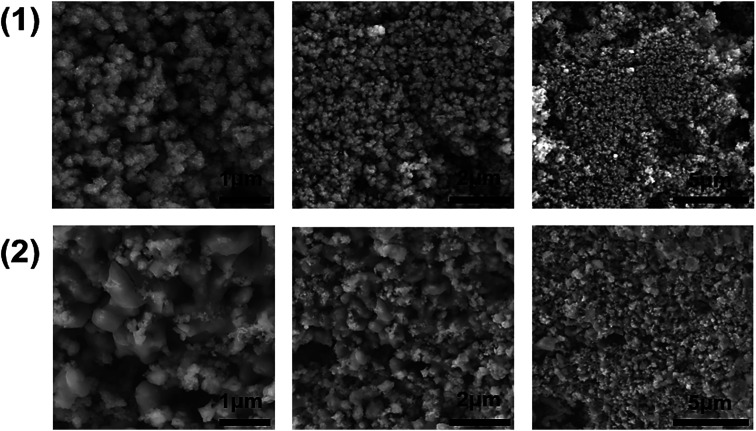
SEM images of Cu_2_FeSnS_4_ powders (1) and (2) synthesised at 450 °C for 1 h taken at different magnifications.

Fig. 5(a–d) show the Fe 2p, Cu 2p, Sn 3d and S 2p X-ray photoelectron spectra recorded from powders prepared using a Sn(ii) and Sn(iv) precursor. The Sn 3d spectra show no difference between the two samples, with peaks at 486.9 eV and 495.3 eV arising from the spin orbit split 3d_5/2_ and 3d_3/2_, respectively. It is difficult to determine the Sn oxidation state from the Sn 3d XPS spectrum since the literature reports both Sn(ii) and Sn(iv) compounds with binding energies in the region. It is possible there is some surface oxidation for both synthesis methods. The S 2p spectra in Fig. 5(d) are fitted with three spin orbit split doublets from S 2p_3/2_ and S 2p_1/2_. Both samples show significant surface oxidation with a substantial sulphate derived peak with the S 2p_3/2_ at a binding energy of 168.8 eV. There is a clear sulphide derived doublet with the 2p_3/2_ at a binding energy of 161.6 eV and some residual contamination at the surface attributed to S–O, S–H or S–C at the surface.^54^ We found that the Fe 2p and Cu 2p spectra in Fig. 5(a and b) are difficult to fit. The d-electrons in these transition metals lead to a range of multiplet split features, and complex shake up structures.^55,56^ Simple analysis of the binding energies of the features in the Cu 2p_3/2_ region are consistent with the presence of CuO at the surface. The strong, narrow peak at a binding energy of 932.2 eV, however, is similar to that found in chalcopyrite (CuFeS_2_) and is more intense than would be expected for CuO.^56^ The Fe 2p spectra also suggest some oxidation at the surface. The binding energy of the lowest energy peak of 711.7 eV is often attributed to the presence of FeSO_4_ and Fe_2_O_3_. The former is consistent with the binding energy of sulphate derived S.^55^ The satellite feature at a binding energy of 725.22 eV is 2p peak in Fig. 6(d) and the lower energy satellite at 716.5 indicative of the presence of Fe(iii) seemingly confirming the oxidation of Fe to Fe_2_O_3_. It is clear that the high binding energy satellite and the 711.7 eV peaks are much more pronounced for the material synthesized from the Sn(iv) precursor, but the reasons for this are unclear. Unfortunately, a lack of high quality XPS spectra from FeCuSnS_2_ standards means it is difficult to determine contributions from this material.

### Microscopic characterisation of CFTS powders

**Fig. 7:**
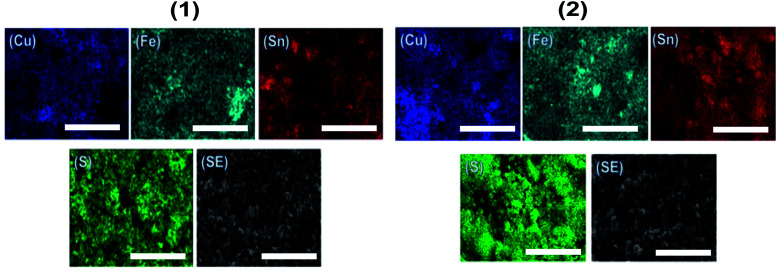
EDX elemental mapping of Cu_2_FeSnS_4_ powders (1) and (2) synthesised at 450 °C for 1 h showing the spatial distribution of Cu, Fe, Sn and S. Scale bars represent 5 μm in all cases. A secondary electron SEM image of the mapped area is included in each case, labelled as SE.

Scanning electron microscopy (SEM) images of the CFTS powders at different magnifications are shown in Fig. 6. Images of CFTS (2) show that the crystallites were largely agglomerated with variation in their size, while agglomerated but less polydisperse particles are obtained in CFTS (1). In both cases agglomeration is random with no ordering of particles observed. The compositional data and EDS spectra of CFTS powder synthesized at 450 °C are shown in Fig. S1.† The atomic % of Cu, Fe, Sn^2+^ and S were 27.76, 13.19, 13.73 and 45.32, respectively in (1), while in (2) the atomic % of Cu, Fe, Sn^4+^ and S were 25.41, 14.09, 15.40 and 45.10, respectively which indicates that both Cu_2_FeSnS_4_ powders have the required stoichiometry. Elemental mapping of CFTS is used to investigate the spatial homogeneity in terms of elemental distribution at the microscale. Fig. 7 shows the elemental mapping CFTS powders. It seems likely based on these images that the distributions of Cu, Fe, Sn and S elements in the sample are uniform at the microscale.

### Optical and electronic properties

**Fig. 8:**
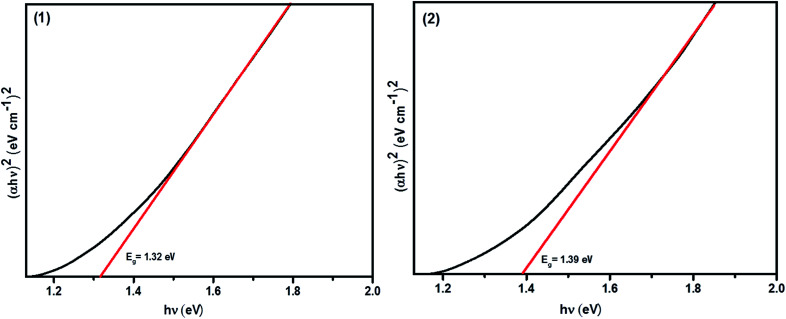
Tauc plot (*ahν*)^2^*vs. hν* showing the direct bandgaps of Cu_2_FeSnS_4_ powders (1) and (2) and their energies.

The UV-Vis-NIR absorbance spectra of the CFTS powders dispersed in ethanol in the wavelength range of 400–1100 nm are shown in Fig. S2.†Fig. 8 shows Tauc plots of (*ahν*)^2^*versus hν* with a straight line fitting, indicating the direct bands gaps of 1.32 eV and 1.39 eV for (1) and (2), respectively, which are in good agreement with literature values.^29,57^ Ideally, the absorber material of a thin film solar cell should be a direct bandgap semiconductor because of the strong optical transitions between the energy bands and high absorption coefficient (*α* > 10^4^ cm^−1^). The calculated limiting efficiency for a single band gap solar cell of *E*_g_ = 1.3–1.4 eV in a simulated solar spectrum (AMG 1.5, *i.e.* fixed incident light) is around 30%. Hence, the optical properties that we measure for these materials suggest that they may well be useful for applications in the absorber layers in solar cells. We therefore studied the electronic properties of these materials as thin films deposited using spin coating. Four-probe Hall measurements performed on CFTS thin films revealed that the majority carriers are holes (p-type), whilst the carrier mobility ranged between 58–60 cm^2^ V^−1^ s^−1^. The estimated carrier densities in these films are of the order of 10^14^ cm^−3^. Full details of the CFTS thin film preparation, characterisation and electronic measurements are given in the ESI.†

## Conclusions

Copper, iron and tin *O*-ethylxanthate complexes have been successfully synthesized. The complexes were found to decompose in the temperature range of 150–450 °C to give the metal sulphide as the final product in conformity with the mass loss data and were used for the synthesis of CFTS powders. The CFTS powder (1) and (2) have been successfully synthesised from both Sn(ii) and Sn(iv) precursors respectively using pyrolysis in the temperature range of 250 to 450 °C. The stannite phase is obtained for both CFTS powders, which was ascertained from a tetragonal distortion parameter *c*/2*a* of less than 1 in all cases. Absorption measurements confirm that Cu_2_FeSnS_4_ powder (1) and (2) are direct band gap semiconductors having bandgap energies of 1.32 eV and 1.39 eV, respectively and thus are suitable for photovoltaic absorber layer applications.

## Conflicts of interest

There are no conflicts of interest to declare.

## Supplementary Material

RA-009-C9RA02926E-s001
