# Supplementary material for: A molecular precursor route to quaternary chalcogenide CFTS (Cu2FeSnS4) powders as potential solar absorber materials
Source: RSC Adv. 2019 Aug 5;9(42):24146–53. doi: 10.1039/c9ra02926e (PMC9069629; doi:10.1039/c9ra02926e)
Supplement: RA-009-C9RA02926E-s001 [file RA-009-C9RA02926E-s001.pdf]

Electronic Supplementary Material (ESI) for RSC Advances.
This journal is © The Royal Society of Chemistry 2019

A molecular precursor route to quaternary chalcogenide CFTS (Cu_2FeSnS_4) powders as potential solar absorber materials

Abdulaziz M. Alanazi,[a,d] Firoz Alam,[a, b] Abdelmajid Salhi,[c] Mohamed Missous,[c] Andrew G. Thomas,[b] Paul O'Brien[a,b] and David J. Lewis.[b]*

[a] School of Chemistry, University of Manchester, Oxford Road, Manchester, M13 9PL, UK.

[b] School of Materials, University of Manchester, Oxford Road, Manchester, M13 9PL, UK.

[c] School of Electrical and Electronic Engineering, University of Manchester, Sackville Street, Manchester, M13 9PL, UK

[d] School of Chemistry, Islamic university, Prince Naif Ibn Abdulaziz Rd, Madinah, 42351, KSA

Corresponding author: E-mail: david.lewis-4@manchester.ac.uk

Supporting Information:

Section: Powder

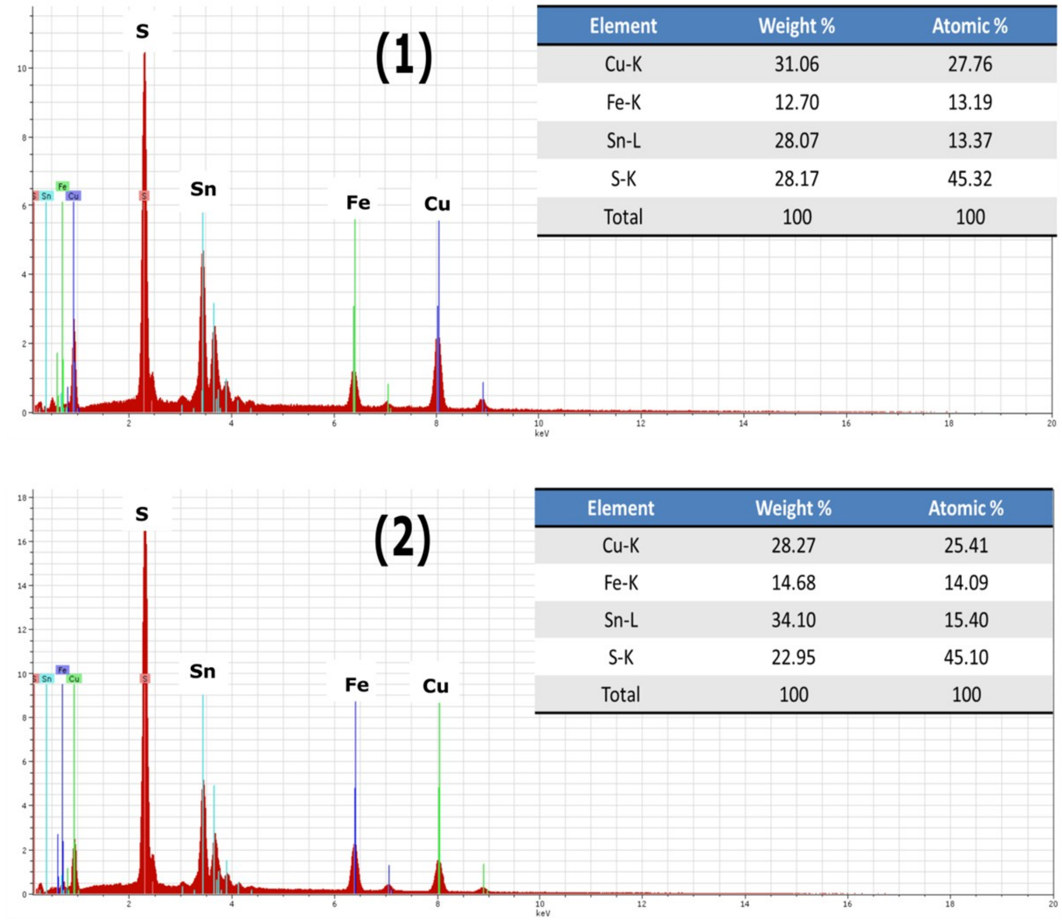

(1)

Element	Weight %	Atomic %
Cu-K	31.06	27.76
Fe-K	12.70	13.19
Sn-L	28.07	13.37
S-K	28.17	45.32
Total	100	100

(2)

Element	Weight %	Atomic %
Cu-K	28.27	25.41
Fe-K	14.68	14.09
Sn-L	34.10	15.40
S-K	22.95	45.10
Total	100	100

Figure S1: The EDX plots of Cu_2FeSnS_4 powder **(1)** and **(2)** synthesised at a temperature of 450°C for 1 hour**.** The inset of Fig showing the compositional data of Cu_2FeSnS_4 powder **(1)** and **(2)**.

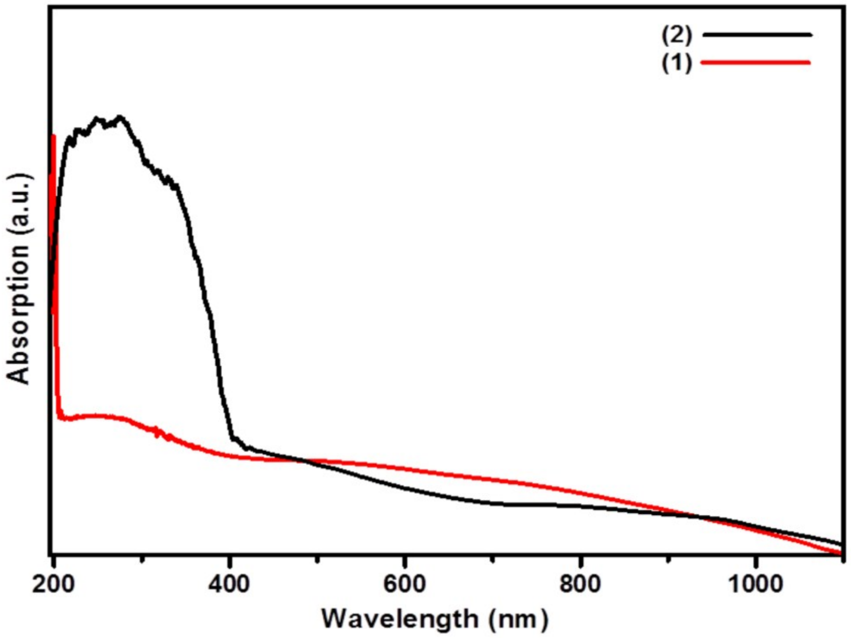

Figure S2: The UV-Vis-NIR absorbance spectra of Cu_2FeSnS_4 powder **(1)** and **(2)** synthesised at a temperature of 450°C for 1 hour.

Section: Thin film

The deposition of CFTS thin films using spin coating technique

Glass substrates were cut to 20 mm × 15 mm, and cleaned by acetone and water and allowed to dry. The solutions were prepared by dissolving the mixture of 2 mmol triphenylphosphine copper(I) ethylxanthates, 1 mmol iron(III) ethylxanthates and 1 mmol tin (II) ethylxanthates or tin (IV) ethylxanthates in tetrahydrofuran (THF, 6 ml). A clear black solution was obtained. The solution was used to deposit CFTS thin films on cleaned glass substrates using spin coating techniques (Ossila, 24 V DC, 2.01 A) at 700 rpm for 30 s and allowed to dry. The resulting films were placed into a tube furnace and heated at 450 ^{o}C for 60 min, under an inert atmosphere. After that the furnace was turned off and the tube was allowed to cool down to room temperature. The CFTS thin films deposited from Sn(II) and Sn(IV) are named as **(3)** and **(4)**, respectively.

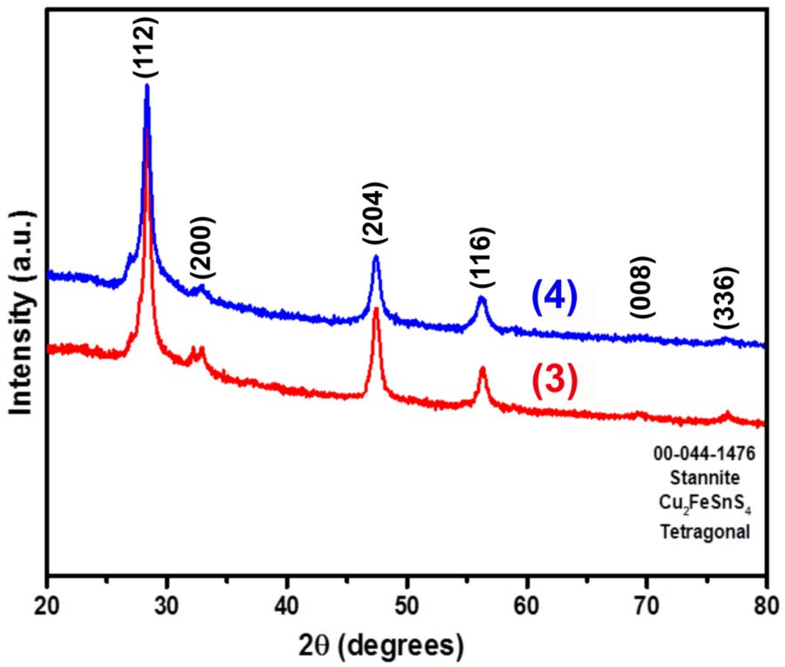

Figure S3: The p-XRD patterns of Cu_2FeSnS_4 thin films deposited using **(3)** and **(4)** and annealed at 450°C for 1 hour.

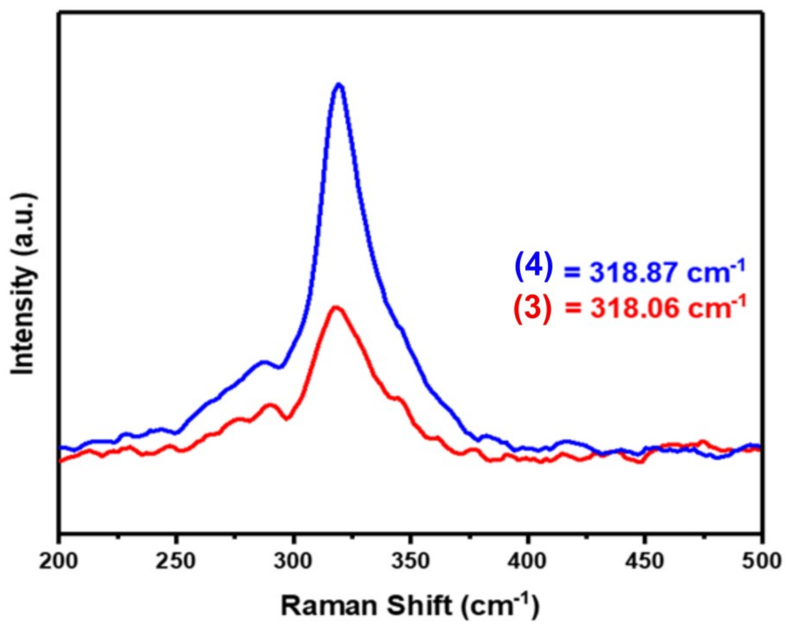

Figure S4: The Raman spectra of Cu_2FeSnS_4 thin films deposited using **(3)** and **(4)** and annealed at 450°C for 1 hour.

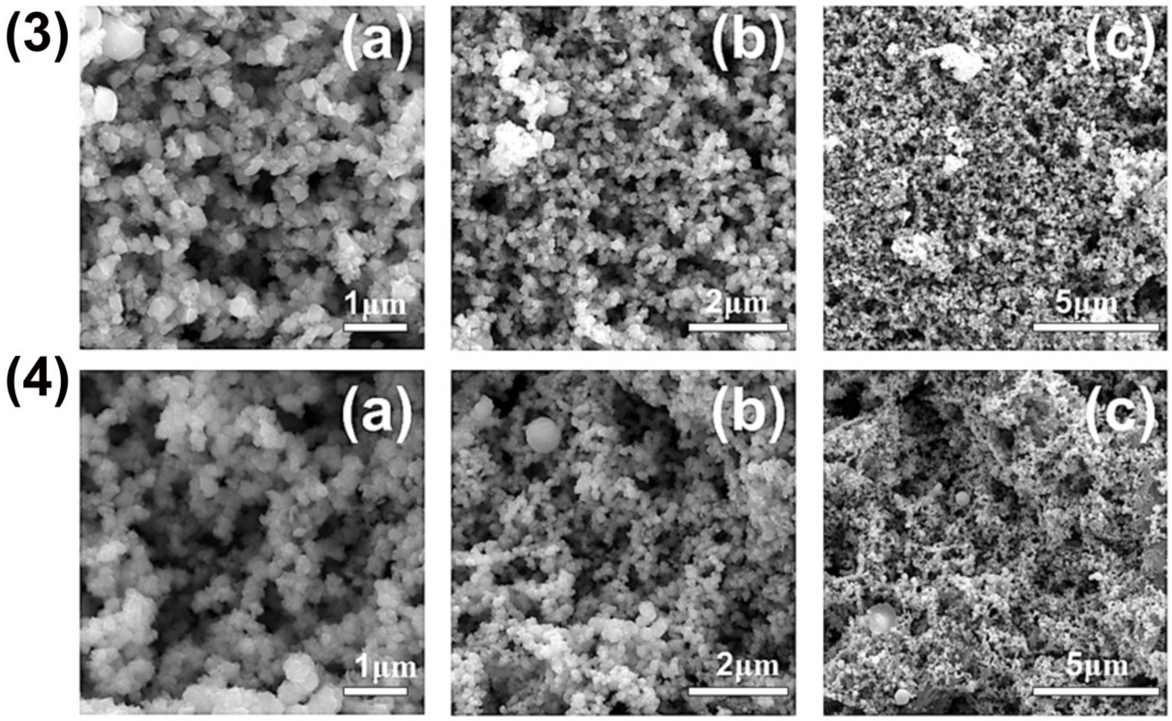

Figure S5: SEM images of Cu_2FeSnS_4 thin films deposited using **(3)** and **(4)** and annealed at 450°C for 1 hour.

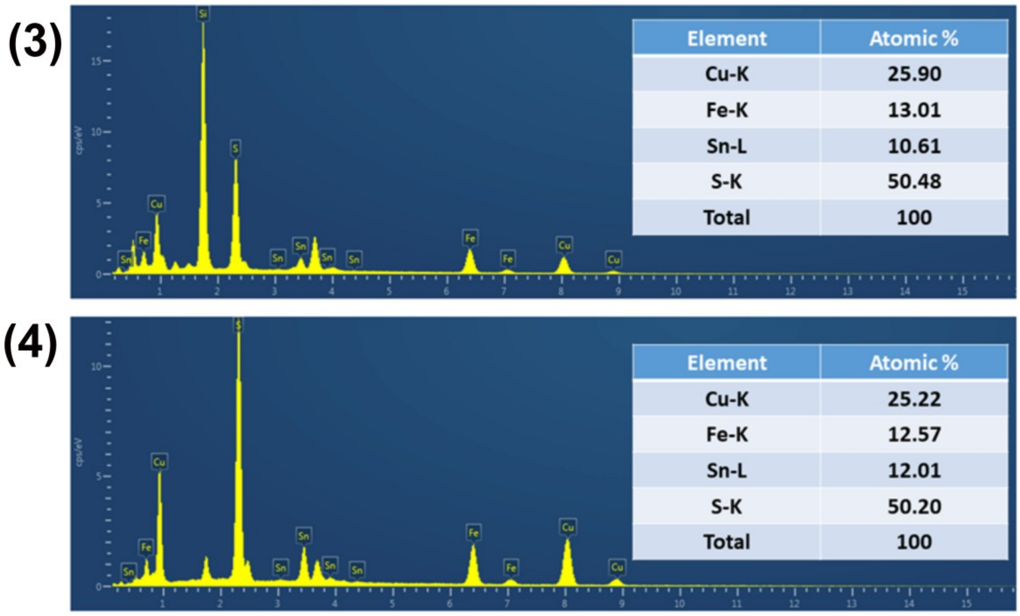

Figure S6: The EDX plots of Cu_2FeSnS_4 thin films deposited from **(3)** and **(4)** and annealed at a temperature of 450°C for 1 hour. The inset image showing the atomic percent of Cu_2FeSnS_4 thin films.

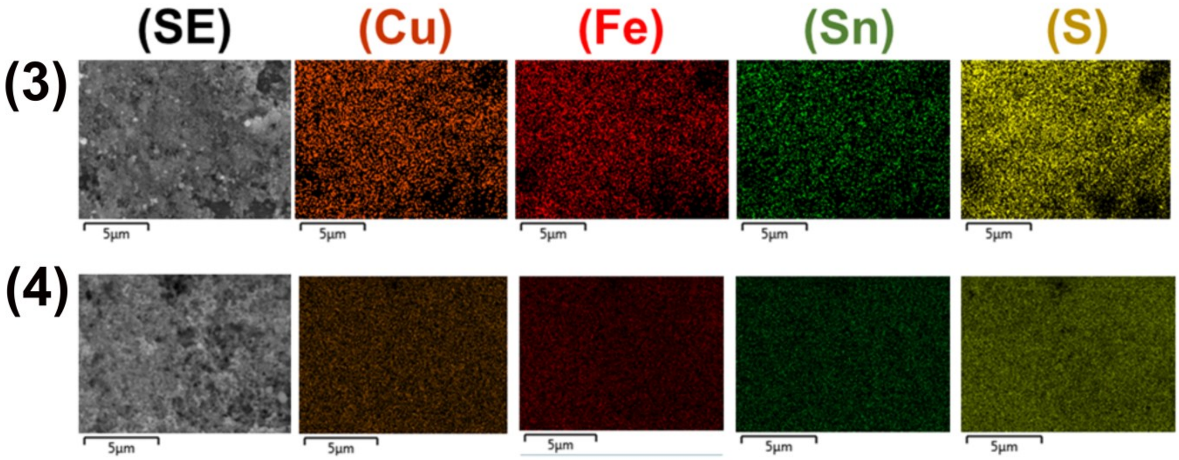

Figure S7: Elemental mapping of Cu_2FeSnS_4 thin films deposited from **(3)** and **(4)** and annealed at 450°C for 1 hour, showing the distribution of Cu, Fe, Sn and S.

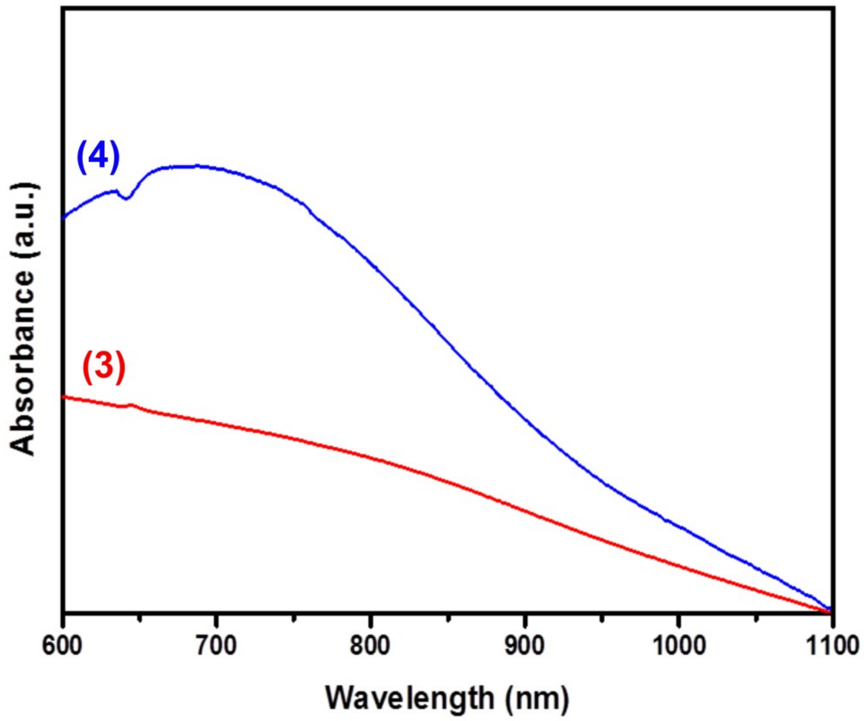

Figure S8: The UV-Vis-NIR absorbance spectra of Cu_2FeSnS_4 thin films deposited from **(3)** and **(4)** and annealed at 450°C for 1 hour.

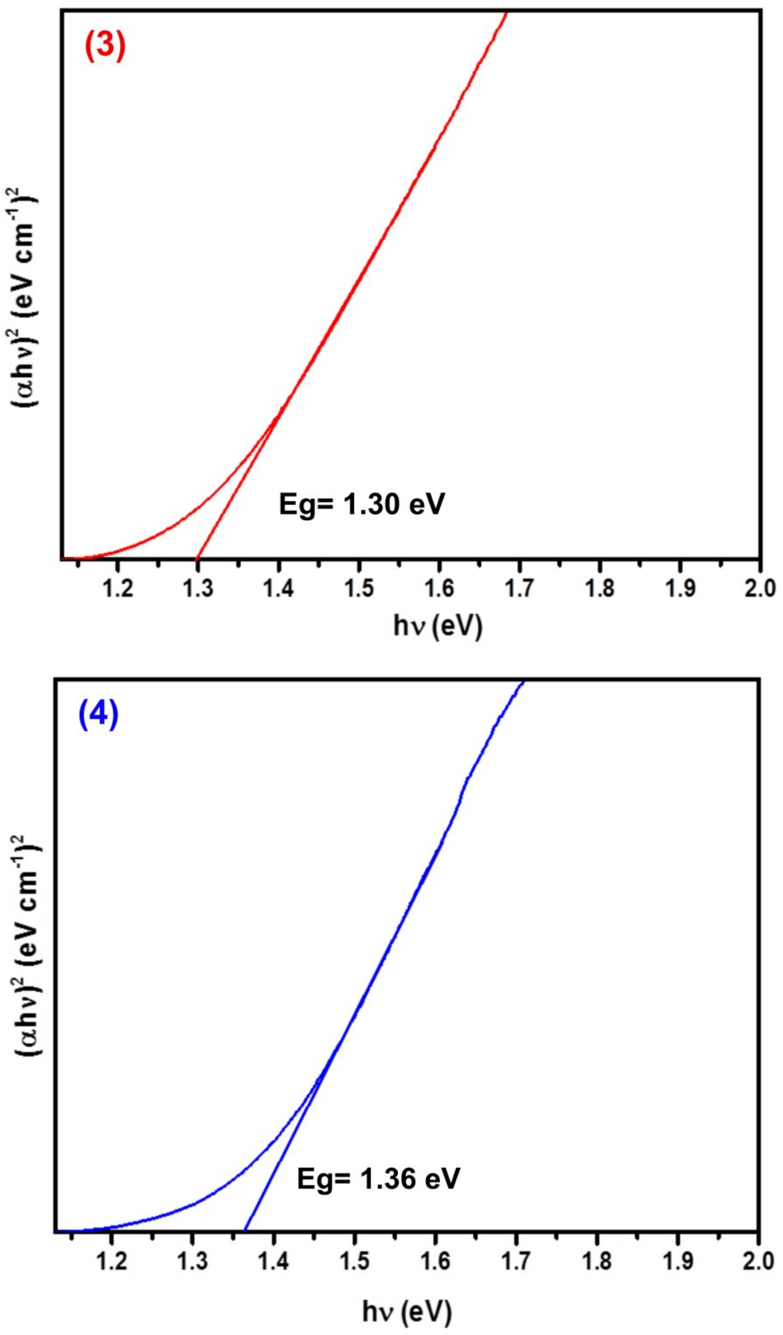

Figure S9: Tauc plot $(\alpha h\nu)^2$ vs. hν showing the direct bandgap of Cu_2FeSnS_4 thin films deposited from **(3)** and **(4)** and annealed at 450°C for 1 hour.

Electrical properties of CFTS thin films

The electrical properties of the CFTS thin films were characterized using Hall measurement in a four-probe configuration. Conductive silver paste was used to form the four contact electrodes. The Hall measurements performed on both CFTS samples of dimension 7mm×7mm shows that the majority carriers are holes, indicating the p-type conductivity in the CFTS films deposited using **(3)** and **(4)**. The carrier mobility in CFTS thin films obtained from **(3)** and **(4)** are 58 $cm^2/V.s$ and 60 $cm^2/V.s$, respectively. The estimated carrier densities are 8.2×10^{14} cm^{-3} and 4.6×10^{14} cm^{-3} for CFTS thin films obtained from **(3)** and **(4)**, respectively. The same finding for CFTS samples have been reported by Prabhakar et al.[1] The obtained values of hole mobility and carrier density suggest that CFTS could be a potential material for photovoltaic applications.[2]

References

1. R.R. Prabhakar, N.H. Loc, M.H. Kumar, P.P. Boix, S. Juan, R.A. John, S.K. Batabyal,L.H. Wong, *ACS Appl. Mater. Interfaces*, 2014, **6**, 17661–17667.
2. D. B. Mitzi, O. Gunawan, T. K.Todorov, K. Wang, S. Guha, *Sol. Energy Mater. Sol. Cells,* 2011, **95**, 1421-1436.
